# Complete Genome Sequence of Strain AC001, a Novel Cultured Member of the Human Oral Microbiome from the Candidate Phylum *Saccharibacteria* (TM7)

**DOI:** 10.1128/MRA.01158-19

**Published:** 2019-10-17

**Authors:** Andrew J. Collins, Pallavi P. Murugkar, Floyd E. Dewhirst

**Affiliations:** a The Forsyth Institute, Cambridge, Massachusetts, USA; b Department of Oral Medicine, Infection and Immunity, Harvard School of Dental Medicine, Boston, Massachusetts, USA; Indiana University, Bloomington

## Abstract

Strain AC001 is a cultured representative of human microbial taxon 488, a bacterium from the candidate phylum *Saccharibacteria*. It is an obligate parasite with a genome of <0.9 Mb and grows in coculture with its host, Pseudopropionibacterium propionicum. The complete genome sequence is presented here.

## ANNOUNCEMENT

**FIG 1:**
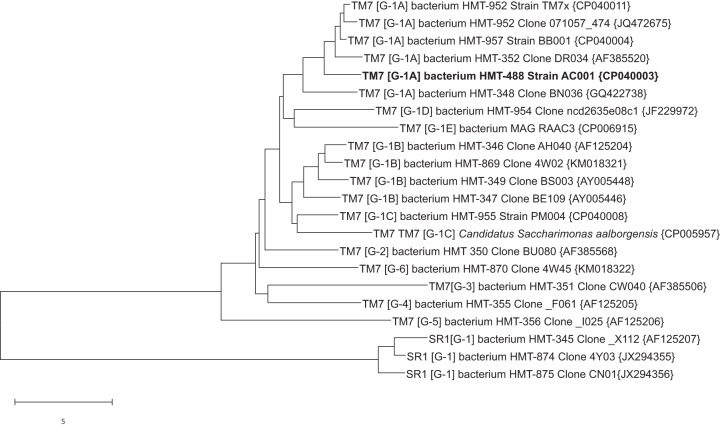
Neighbor-joining tree (10) for *Saccharibacteria* (TM7) bacterium HMT 488 strain AC001. The tree was produced from aligned full-length 16S rRNA sequences (∼1,450 bp) using MEGA X (11). The designations in square brackets are the TM7 class level groups as described in reference 12. GenBank accession numbers for 16S rRNA or genome sequences are given in curly brackets. The scale bar shows a 5% difference in sequence similarity. For trees showing broader TM7 diversity, see references [Bibr B12][Bibr B13][Bibr B14].

Twenty-five percent of bacterial species on earth are thought to be members of the candidate phyla radiation (CPR) (1, 2). A human oral cavity isolate from the candidate phylum *Saccharibacteria*, TM7x, was the first species to be cultured from the CPR (3, 4). The bacterium was isolated in coculture with Actinomyces odontolyticus strain XH001 and is thought to be an obligate parasitic epibiont (5, 6). Strain TM7x is a member of human microbiome taxon (HMT) 952 in the Human Oral Microbial Database (7, 8). Strain AC001 was isolated in coculture with Pseudopropionibacterium propionicum strain F0700. The HMT 488 strain AC001 differs sufficiently from HMT 952 strain TM7x by both 16S rRNA (97.5% identity) and genome comparisons to be considered a novel species in the same genus. A phylogenetic tree for human oral and important environmental *Saccharibacteria* is presented in Fig. 1. The strain AC001 was isolated in Cambridge, MA, from the dental plaque of a healthy 31-year-old white male. The sample was dispersed and filtered through a 0.2-μm filter to remove all but ultrasmall bacteria. The filtrate was added to broth cultures of several human oral species, and a novel *Saccharibacteria* species was recovered growing in coculture with host bacterium P. propionicum.

For DNA isolation, strain AC001 was cocultured with *P. propionicum* in 200 ml of a 50/50 blend of Trypticase soy and brain heart infusion broth with 1% yeast extract. The culture was filtered through a 0.45-μm filter which retained *P. propionicum* and passed the much smaller *Saccharibacteria* cells, which were then pelleted by centrifugation. Genomic DNA was isolated using a modified MasterPure DNA isolation kit (Epicentre) protocol, which included a bead-beating step to increase cell lysis. Library preparation and DNA sequencing were performed at the Johns Hopkins Deep Sequencing and Microarray Core. Genomic DNA was sheared to 10 to 20 kb using a Covaris g-TUBE and purified using AMPure XP beads (Agencourt Bioscience). Size selection and further cleanup were performed using BluePippin (Sage Science). The library was sequenced on a PacBio RS II instrument on one single-molecule real-time (SMRT) cell per library. Default parameters were used for all software. The reads were assembled using the SMRT Analysis software version 2.3.0 HGAP3 pipeline. Methylation motifs were detected using the SMRT Analysis software version 2.3.0 Base_Modification_and_Motif_ Analysis pipeline. Genes were annotated using the NCBI Prokaryotic Genome Annotation Pipeline using the best-placed reference protein set (GeneMarkS-2+ v4.8) (9).

There were 60,281 raw reads covering 785,422,154 sequenced bases. The mean read length was 13,029 bases, and the *N*_50_ read length was 21,543 bases. The average reference coverage was 728×. The genome assembled into a single contig of 936,284 bp and was circularized to 889,964 bp by removing a duplicated 46-kbp segment at each end of the initial contig. The GC content of the DNA was 50.6%. Genome annotation identified a total 942 genes, of which 885 were predicted to be coding sequences (CDSs), 49 were RNAs, and 8 were pseudogenes.

### Data availability.

The genome sequence was deposited at GenBank under accession number CP040003 and SRA accession number SRR9734869. Base modification files were submitted with the GenBank accession.
